# Influence of Wind Speed on CO_2_ and CH_4_ Concentrations at a Rural Site

**DOI:** 10.3390/ijerph18168397

**Published:** 2021-08-09

**Authors:** Isidro A. Pérez, María de los Ángeles García, María Luisa Sánchez, Nuria Pardo

**Affiliations:** Department of Applied Physics, Faculty of Sciences, University of Valladolid, Paseo de Belén, 7, 47011 Valladolid, Spain; magperez@fa1.uva.es (M.d.l.Á.G.); mluisa.sanchez@uva.es (M.L.S.); npardo@fa1.uva.es (N.P.)

**Keywords:** METEX, skewed distributions, index of agreement, Nash–Sutcliffe efficiency, daily cycle, annual cycle

## Abstract

Meteorological variables have a noticeable impact on pollutant concentrations. Among these variables, wind speed is typically measured, although research into how pollutants respond to it can be improved. This study considers nine years of hourly CO_2_ and CH_4_ measurements at a rural site, where wind speed values were calculated by the METEX model. Nine wind speed intervals are proposed where concentrations, distribution functions, and daily as well as annual cycles are calculated. Contrasts between local and transported concentrations are around 5 and 0.03 ppm for CO_2_ and CH_4_, respectively. Seven skewed distributions are applied, and five efficiency criteria are considered to test the goodness of fit, with the modified Nash–Sutcliffe efficiency proving to be the most sensitive statistic. The Gumbel distribution is seen to be the most suitable for CO_2_, whereas the Weibull distribution is chosen for CH_4_, with the exponential function being the worst. Finally, daily and annual cycles are analysed, where a gradual decrease in amplitude is observed, particularly for the daily cycle. Parametric and nonparametric procedures are used to fit both cycles. The latter gave the best fits, with the agreement being higher for the daily cycle, where evolution is smoother than for the annual cycle.

## 1. Introduction

Cities are major sources of air pollution, and a reduction in pollution levels must be achieved by emission control. Due to its significant environmental impact, the European Union (EU) is seeking to reduce household energy consumption. However, although this consumption decreased over the period 2005–2016 and Spain had the fourth lowest consumption in the EU, this trend might not be maintained, since energy efficiency improvements may be offset by lifestyle- or weather-related changes [1].

Moreover, meteorological conditions play a key role in the measured concentrations. Dong et al. [2] analysed air pollution in the Beijing-Tianjin-Hebei region of China, one of the most populated regions, comparable to the Pearl River Delta or the Yangtze River Delta regions, and reported that the impact of meteorology on air pollution ranges from 20% to 40%, depending on the type of process, i.e., whether there is local or regional transport. Mousavinezhad et al. [3] studied the ozone trend at these three sites and concluded that changes in meteorological variables, such as solar radiation, temperature, and sea level pressure, were associated with ozone changes of around 32% in the Beijing-Tianjin-Hebei region. Synoptic types have usually been considered as a suitable procedure to link air pollution and meteorology [4]. However, other analyses address this relationship by considering individual meteorological variables [5].

Some studies focus on specific variables. One such case is the analysis by Miao et al. [6], which investigates the role of the planetary boundary layer on air pollution in Beijing and Shanghai. Although the influence of wind speed on air pollution is recognised, studies exclusively devoted to this variable are scarce. Wind speed is linked with both local and transboundary pollution episodes. One example is presented by Yoshino et al. [7], who analysed air quality at Fukuoka, Japan, and established two wind speed groups with a 3 m·s^−1^ frontier. Wind speeds below this value were associated with varied wind directions, whereas wind speeds above 3 m·s^−1^ yielded nearly straight air parcel trajectories. A slightly more elaborate treatment includes wind speed intervals and even wind direction intervals [8].

To date, direct relationships between CO_2_ concentrations and wind speed have been considered and even parameterised with exponential functions [9]. García et al. [10] employed such equations for concentrations measured at a rural site and reported results that agreed with similar studies. The most noticeable inconvenience of these analyses is the pronounced scatter of measurements where accused outliers highlight and mask the most frequent concentration pattern. The current research overcomes this drawback by using statistics calculated in wind speed intervals. This is the aim of the first part of this paper.

The second part of this study focuses on the distribution functions. These functions are required to provide an easy description of large amounts of observations. One additional feature is that they may be used to interpolate and extrapolate observations by probability calculation in certain ranges. Moreover, since the functions used to fit the data are smooth, irregularities associated with specific observations are avoided [11].

Symmetric distribution functions have occasionally been used for particulate matter [12,13]. However, skewed distribution functions offer a wider field of application, where symmetric distributions are particular types. Concentrations of certain air pollutants, such as ozone, have been described by skewed distributions [14,15,16].

CO_2_ concentration distribution has already been analysed by means of skewed distributions [17]. However, the influence of wind speed on such distributions remains an open field of study. This paper expands this research line by including CH_4_ concentrations. Moreover, varied efficiency criteria are used to select the most suitable distribution function and to compare the efficiency criteria themselves.

Finally, the last part of this analysis considers the daily and annual cycles of CO_2_ and CH_4_. Although these cycles have previously been described [18,19], their response to wind speed merits a detailed study. Two procedures, one parametric and another nonparametric, are used to smooth and investigate the two cycles. The main advantage of parametric procedures is the closed form of expressions, which may be easily determined. However, the response of nonparametric procedures may prove to be better for specific intervals, although the calculation may be slower than for parametric procedures.

## 2. Materials and Methods

### 2.1. CO_2_ and CH_4_ Observations

Dry concentrations of both gases were measured with a Picarro G1301 analyser in the centre of the northern plateau of the Iberian Peninsula (CIBA station, 41°48′50″ N, 4°55′59″ W, 852 m a.s.l.). The analysis period covered nine years from October 2010. Although observations were obtained at three levels, only the highest (at 8.25 m a.g.l.) was used in this paper, and hourly averages were calculated.

**Figure 1:**
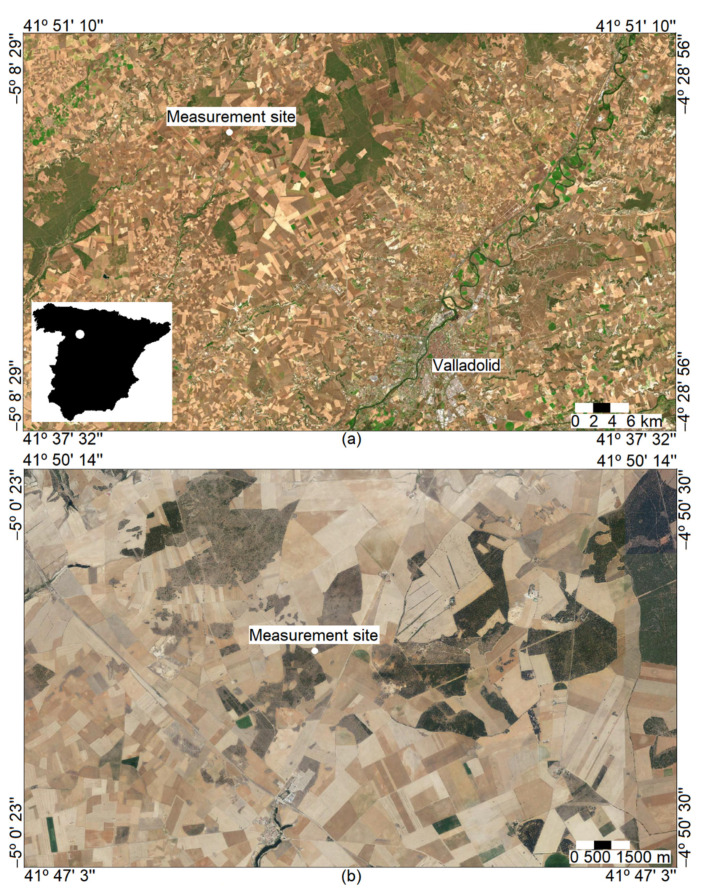
(**a**) Location of the measurement site and the main urban site, Valladolid. Bottom left is the location of the measurement site in Spain. (**b**) Surrounding area of the measurement site, formed mainly by crops. Images courtesy of © ign.es.

The measurement site is nearly flat and has no orographic features in its surrounding area. Figure 1 presents the measurement site, which is located in a scrubland area. Landscape is agricultural, and is mainly formed by rainfed crops, and scattered trees are also present. The main nearby city is Valladolid, which lies some 25 km from the measurement site.

### 2.2. Wind Speed

These data were obtained from the METEX model [20]. A 10 m a.g.l. height was considered since this level is common in meteorological analyses. Although varied wind speed classifications have been suggested following different applications, this paper considers one simple procedure with nine classes. The interval width is 1 m·s^−1^ until the eighth class, with the last corresponding to wind speeds above 8 m·s^−1^.

### 2.3. Theoretical Distributions and Distribution Fitting

Since outliers make the distribution shape different from the Gaussian, seven skewed distributions were used. These are presented in Table 1. Although nonparametric procedures guarantee satisfactory fits of histograms, certain parametric expressions may be directly fitted and are preferred, since numerical iterations are not required. Moreover, they are flexible enough to successfully describe the observations, have already been theoretically developed, and the application to gas concentrations extends their field of use.

Additionally, distribution agreement with the observations is compared using certain statistics presented in Table 2, where *O* corresponds to the observed values, and *C* to the calculated ones, with the two following objectives. The first is to select the distribution that achieves the best agreement, and the second is to compare the response of the statistics used in order to choose the most sensitive. One of these statistics, the correlation coefficient, is frequently used, although satisfactory values of this coefficient are reached with over- or under-predictions of calculations. However, the rest are statistics that were proposed in order to overcome the restrictions of the correlation coefficient [24].

### 2.4. Daily and Annual Cycles

These cycles were described by two statistics each hour and each month. The first is the mean, which may be affected by outliers, and the second is the median, which is more robust. Small differences between them reveal a regular distribution of observations whereas a contrast between the mean and the median indicates irregularly placed outliers that may skew the distribution. Moreover, the description of the two cycles was modelled by two procedures. The first is parametric, since it is based on the addition of two harmonic functions, following the equation

(1) $$y=a_{0}+a_{1}cos\left(\frac{2\pi }{T}t-\theta_{0}\right)+a_{2}cos\left(\frac{4\pi }{T}t-\theta_{1}\right),$$

where *y* is the concentration, *t* is time, and the period of the second harmonic function is the half period of the first harmonic function. The difference between the constants *θ*_0_ and *θ*_1_ allows the symmetry of the first harmonic function to be broken. Both constants, together with the rest of the coefficients *a*_i_, may be calculated by multiple linear regression.

A nonparametric method was also considered, with the following expression being used:

(2) $$y\left(t,h\right)=\frac{\sum_{i=1}^{n}K\left(\frac{t-t_{i}}{h}\right)y_{i}}{\sum_{i=1}^{n}K\left(\frac{t-t_{i}}{h}\right)},$$

where the subscript *i* denotes the values known, *h* is the window width, and the Gaussian kernel function was used for the calculations.

(3) $$K\left(x\right)={\left(2\pi \right)}^{-1/2}\exp\left(-0.5x^{2}\right),$$

The statistics presented in Table 2 were considered in order to establish the goodness of the fit.

## 3. Results

### 3.1. Wind Speed and Concentration Distributions

**Figure 2:**
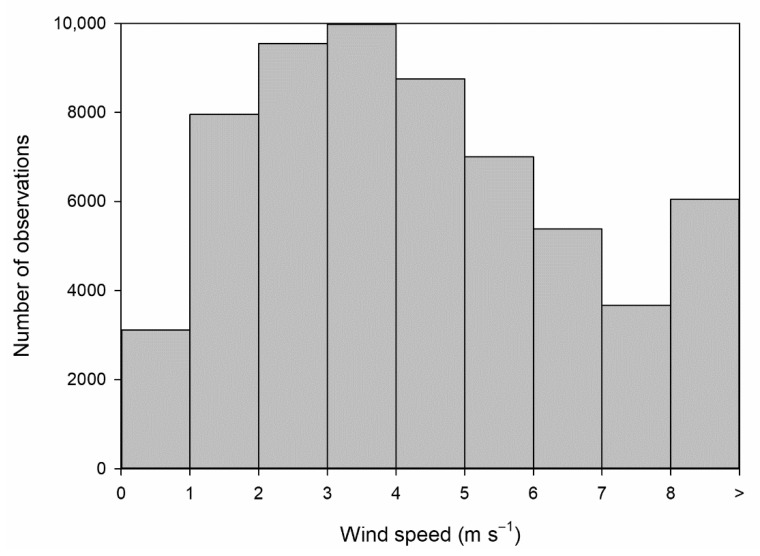
Wind speed distribution.

The wind speed distribution in 1 m·s^−1^ intervals is given in Figure 2, which may be satisfactorily described by the Weibull distribution. This figure corresponds to observations where concentration data were available, i.e., around 78% of the period investigated. Observations above 8 m·s^−1^ are merged in a single class to obtain robust results since outlier analysis lies outside the scope of this paper.

**Figure 3:**
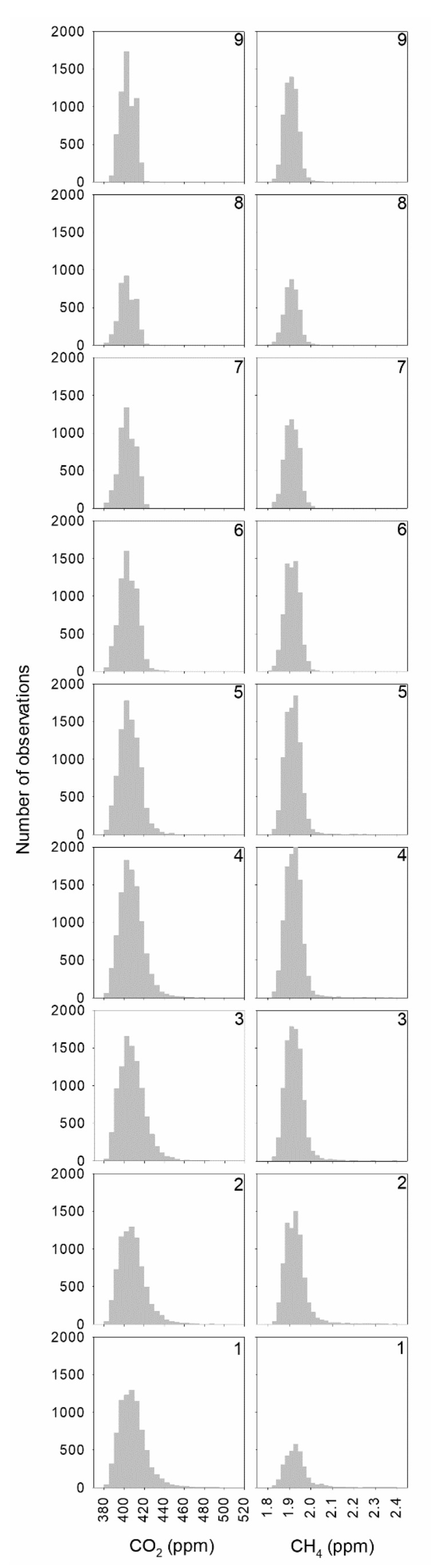
Concentration histograms following the wind speed classification.

**Figure 4:**
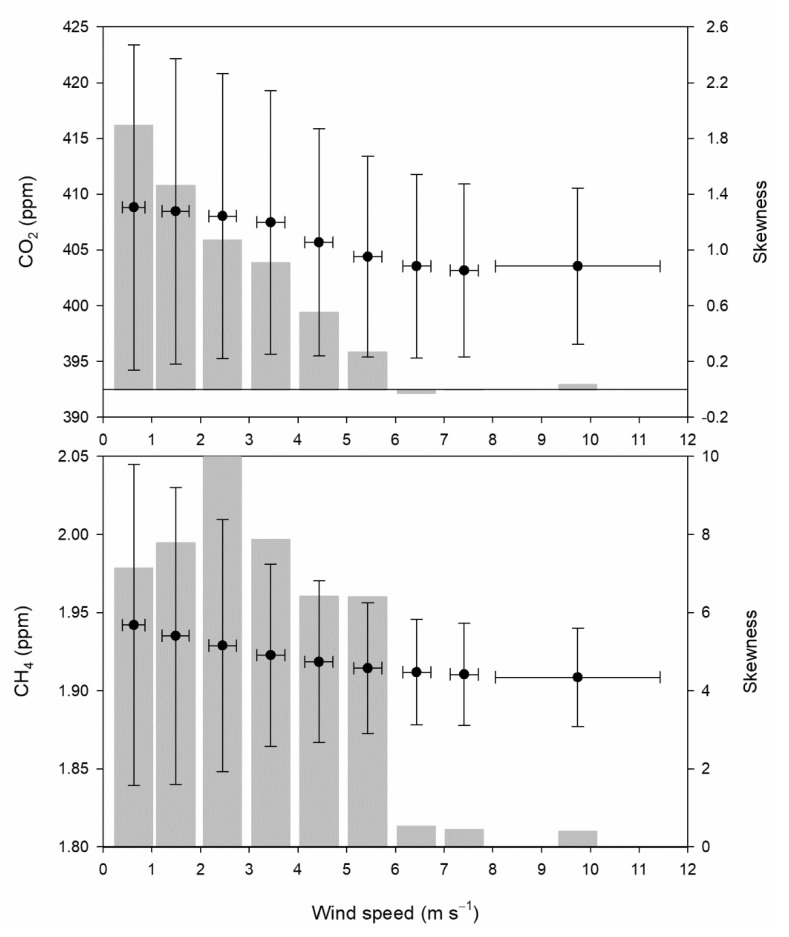
Means, standard deviations, and skewnesses of the concentrations.

Following this wind classification, concentration histograms are presented in Figure 3. A tail on the right is observed for concentrations at low wind speeds for both gases, such as classes below 5 m·s^−1^. However, a small tail on the left is also present at high wind speed, between 6 and 8 m·s^−1^ for CO_2_. Figure 4 presents the means and standard deviations of concentrations and wind speed corresponding to wind speed classes as well as the skewness of concentrations. Two intervals with a slight decrease in CO_2_ concentration were observed: the first for wind speeds below 4 m·s^−1^, with a decrease of around 1.3 ppm, and the second until 8 m·s^−1^, with a decrease of 2.5 ppm. The mean concentration of the last wind speed class is around 0.5 ppm higher than that of the preceding one. Additionally, a gradual decrease in concentration dispersion was observed when wind speed increased. Moreover, skewness also decreased gradually, and even reached negative values in classes 7 and 8. The CH_4_ decrease with wind speed was regular. However, its skewness presented a noticeable contrast since it was high until class 6, with one maximum in class 3, although it was very low for the three classes with the highest wind speed. The origin of concentrations of class 1 could be considered local. However, concentrations of class 9 are influenced by transport and could be taken as background concentrations.

**Figure 5:**
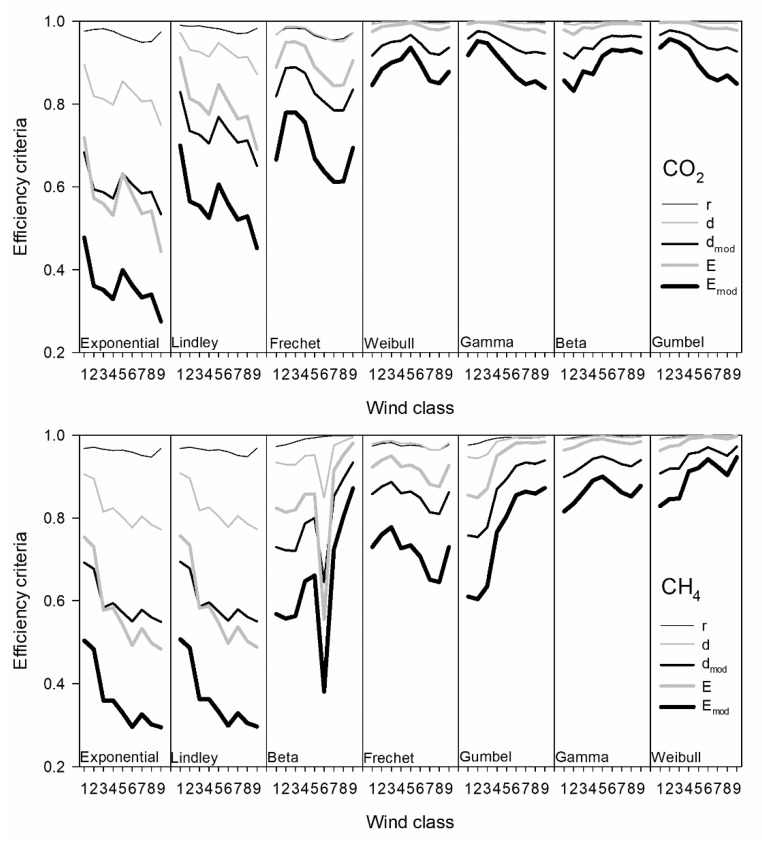
Distribution functions ordered following the efficiency criteria.

Figure 5 presents the efficiency criteria for the distributions used. These were calculated through a comparison between the experimental and the theoretical cumulative distribution functions and were ordered from worst, on the left, to best, on the right. Following these values, the Weibull, Gamma, Beta, and Gumbel distributions offer a similar description for the CO_2_ concentrations, with comparable ranges and shapes of efficiency statistics. The range of the statistics decreased gradually from the exponential to the Frechet distribution. For CH_4_, the best fits were obtained for the Weibull and Gamma distributions, and the worst for the exponential and Lindley distributions. The decrease in the range of statistics is smoother than that of CO_2_. In general, the modified Nash–Sutcliffe efficiency is the statistic with the lowest values, followed by the modified index of agreement. Consequently, both should be used rather than the correlation coefficient, which presents a narrow interval.

### 3.2. Daily and Annual Cycles

**Figure 6:**
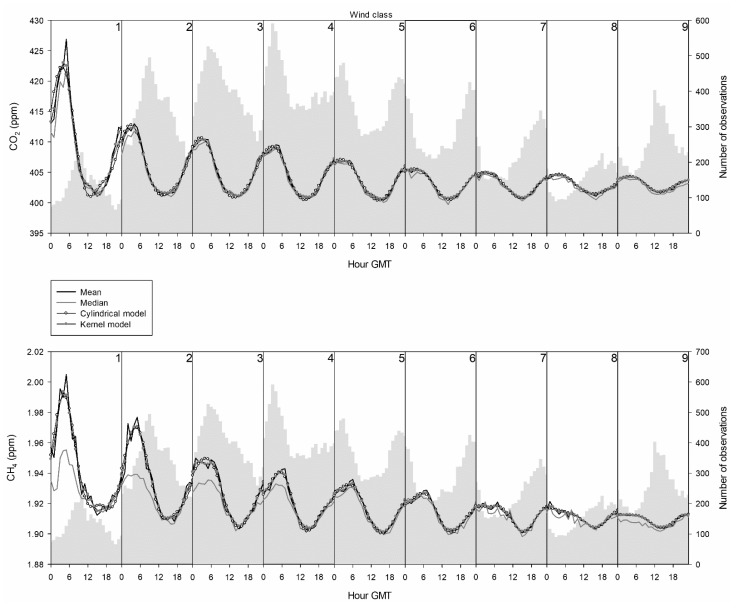
Daily cycle following the wind speed classification.

Figure 6 presents the daily distribution of observations together with the evolution of the median and mean concentrations; this latter one is fitted by the cylindrical and kernel models. A window width, *h*, is required in this last method, although determining it was not the objective of this paper. Consequently, one hour was fixed following observation availability, and a satisfactory agreement was observed. For low wind speeds below 2 m·s^−1^ and for the highest wind speeds above 8 m·s^−1^, the most frequent values are reached at midday. However, at intermediate wind speeds between 2 and 8 m·s^−1^, the highest frequencies are displaced to late night for low wind speeds, early night for high wind speeds, or during the night for wind speeds from 4 to 6 m·s^−1^. Mean, median, and modelled values are similar for CO_2_, with the highest amplitude of the daily cycle and the highest concentrations occurring after midnight for the lowest wind speed. A similar shape is observed for CH_4_, although the contrast between the median and the mean is more noticeable for wind speeds below 3 m·s^−1^, which may be linked to the frequent outliers of high concentrations at such wind speeds.

Table 3 presents the average efficiency statistics when means and modelled values are compared. As in the previous case, when distributions are considered, the modified Nash–Sutcliffe efficiency, followed by the modified index of agreement, achieves the lowest values, and, for both gases, the kernel model fits better than the cylindrical model.

**Figure 7:**
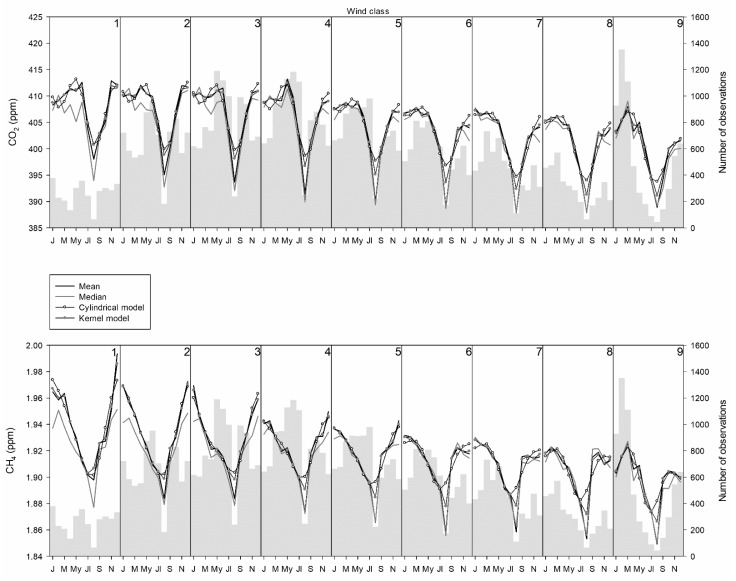
Annual cycle following the wind speed classification.

Figure 7 presents the frequency of observations together with the annual cycle of means, medians, and modelled observations. One month was initially suggested for the window width in the annual cycle with the kernel procedure, and a smooth evolution was observed. Consequently, a narrower window equal to half a month was used. Since the equipment was switched off for most of the months of August, the frequencies observed in this month were low. For wind speed below 1 m·s^−1^, observations are regularly distributed during the year. However, for wind speed from 1 to 3 m·s^−1^, high frequencies are observed in the central part of the year. For wind speeds from 3 to 7 m·s^−1^, high frequencies are obtained in the first half of the year, and, finally, for wind speeds above 7 m·s^−1^, high frequencies are found in the first few months of the year.

Although the lowest concentration of both gases is reached in summer, especially in August, some differences between them are noticeable. Below 5 m·s^−1^, the interval of low concentrations for CO_2_ was narrow, whereas it was prominent in the CH_4_ annual cycle, with a gradual decrease in the first half of the year and a gradual increase after summer.

Similar statistics were used to compare the means and modelled values (Table 4). The pattern of results was similar to that of the daily cycle, with the modified Nash–Sutcliffe efficiency followed by the modified index of agreement being the estimators with the lowest values. As with the distribution fitting, both statistics are sufficiently sensitive to the contrast between the values they evaluate and may be chosen against others that are more frequent, such as the correlation coefficient.

## 4. Discussion

### 4.1. Wind Speed

The region of the measurement site is one of the largest in the EU and has one of the lowest population densities, a population which is decreasing even further [25]. The population is mainly rural, and urbanization is low, with Valladolid (around 300,000 inhabitants to the SE of the site, Figure 1) being the largest city in the region. Duarte et al. [26] indicated that CO_2_ emissions in Spanish households in 1999 were below the country average for a sparse population density (−12.4%) and for rural sites (−11.2%). Consequently, CO_2_ emissions linked with the urban development observed in various Chinese cities [27] may be discarded or considered to have a negligible effect. Even at rural sites in the region, the population is low [28], and its contribution to atmospheric CO_2_ may be considered slight when compared to sites where there is a greater contribution of the rural population [29].

Information about the prevailing wind direction at the site for high wind speed was available from Pérez et al. [30], where measurements at 100 m revealed no prevailing wind directions for wind speeds below 4 m·s^−1^ but two opposite prevailing wind directions for wind speeds between 4 and 15 m·s^−1^, ENE and WSW, where transport is regional and these directions are not affected by nearby pollution sources. Figure 3 indicates that CO_2_ histograms are right-skewed for low wind speed, since noticeable outliers present a local origin. However, distributions are nearly symmetrical or even left-skewed for high wind speeds where prevailing directions are not affected by nearby sources.

The dispersion of values observed in Figure 4 is mainly attributed to the daily evolution presented in Figure 6, since smooth cycles are obtained with high wind speeds, which are linked with low dispersion, whereas cycles with large amplitudes are observed for low wind speeds, which are associated with noticeable dispersion.

The inverse relationship between CO_2_ and wind speed has been suggested by different analyses, such as Al-Bayati et al. [31] for satellite observations in Iraq or Dimitriou et al. [32], who also presented CH_4_ concentrations in the city of Athens. This latter study considers a scatterplot with seasonal concentrations. The main drawback of such a representation is observation overlapping, which makes it difficult to obtain an average concentration for a wind speed interval. One additional feature is that outliers are prominent, although their contribution to the average concentration may be only slight.

Duan et al. [33] proposed six intervals, five with a width of 1 m·s^−1^ and the last one for the remaining wind speeds. Boxplots for CO_2_ concentrations ranged from 300 to 550 ppm, i.e., they were higher than those used in this study. Medians frequently decreased with wind speed, although in spring the initial decrease was followed by a final increase. Moreover, noticeable interquartile ranges were observed for high wind speeds in summer. Boxplots were also used by Pathakoti et al. [34], who analysed the influence of meteorological parameters on CO_2_ at the Indian Antarctic research station. Under these extreme conditions, a width of 5 m·s^−1^ was used and concentrations remained nearly steady below 20 m·s^−1^ and only decreased above this wind speed.

Alternative graphs have been used by different authors. Mai et al. [35] analysed CO_2_ concentrations at the Pearl River Delta region in China with a plot where seven narrow intervals (width of 0.5 m·s^−1^) are used and in which the standard deviation of concentrations is included. The decrease in CO_2_ concentration reached nearly 20 ppm, with similar concentrations below 2 m·s^−1^ in autumn. Wei et al. [36] analysed CO_2_ and CH_4_ concentrations in Shanghai, China, with graphs where box and violin plots were combined. Five wind speed intervals were used with a 1 m·s^−1^ width and one additional interval for wind speeds above 5 m·s^−1^. The decrease in concentration for both gases was noticeable for wind speeds below 4 m·s^−1^, and the increase was slow above this wind speed for CO_2_, whereas concentrations remained steady for CH_4_. In both cases, the relatively high CO_2_ concentrations for high wind speeds were attributed to transport of polluted air masses. The response of CO_2_ concentrations to wind speed was similar in Figure 4, revealing the slight contribution of the surroundings at the measurement site.

### 4.2. Distribution Fitting

The presence of outliers determines skewed distributions. Pérez et al. [37] analysed the skewness of CO_2_ concentration using a range of statistics. Among them, the Yule coefficient was selected following the relationship between skewness and the daily evolution of concentrations. Although global distribution was right-skewed, left-skewed distributions prevailed at midday. Moreover, the influence of wind speed on skewness revealed low, and even negative, values for high wind speeds.

Skewed functions expand the field of application of symmetric functions both in spatial and temporal research. They were used by Pérez et al. [38] in a spatial analysis of CO_2_ concentrations to investigate the impact of the Valladolid urban plume at this rural site by its extension, around 135 degrees, and magnitude, slightly below 10 ppm. Differences among the functions considered included the place of the tail and the flatness of the curve. Various statistics were used in the study to select the cubic function, followed by the gamma and Weibull distributions as the most suitable to fit urban plume concentrations. Moreover, skewed functions were also used by Pérez et al. [39] to investigate the daily cycle of CO_2_ concentrations. In this case, the best fit was obtained with the generalised von Mises function.

Pérez et al. [17] fitted CO_2_ concentration histograms to 14 skewed distributions. Parameter calculation was performed with equations established using analytical procedures in nine of them. However, the rest were obtained by numerical procedures. These are iterative methods that slow down the calculation. The current analysis retains typical distributions and considers certain functions, such as the Frechet and Lindley functions, which have not been used to date. Moreover, parameter calculation is direct, since numerical procedures are avoided.

Perez et al. [38] used 12 statistics to compare calculated and observed concentrations. Moreover, since the ranges of these statistics differ substantially and the response of each equation may be different to each statistic, they were combined into one comprehensive metric proposed by Zhou et al. [40] where each statistic has the same weight. In the current analysis, two of these statistics, the correlation coefficient and the index of agreement, were retained. However, the Nash–Sutcliffe efficiency and the modified forms of the index of agreement and the Nash–Sutcliffe efficiency are statistics that have scarcely been explored in the context of this study.

### 4.3. Fitting of Daily and Annual Cycles

Harmonic functions are usually used to describe the annual evolution of the gases considered in this study. This is a parametric procedure with substantial flexibility since the number of harmonics is variable and usually ranges from the simplest method, with only one harmonic [41], to the most complex one, with four harmonics [42,43].

CO_2_ and CH_4_ annual cycles at remote and rural sites frequently present a minimum in summer attributed to low plant and soil activity [44]. However, the shape of this cycle depends on the site, since even remote places may be affected by pollution transport. This is the case of certain high concentration events recorded at some European high-altitude stations, such as Zugspitze in Germany, attributed to the atmospheric circulation that carries polluted air from the European plains to the Alpine stations [45,46]. Moreover, high CH_4_ values observed in summer at Mount Waliguan, China, revealed the agricultural source, rice growing, of CH_4_ [47].

Parameterisation of these cycles should be adapted to each measurement station. However, even for the simplest cycles, the one harmonic evolution may be considered as an initial approximation, since a six-month period between the maximum and the minimum is difficult to achieve [48,49]. Pérez et al. [19] tried different combinations of four harmonics and recommended using the first and second harmonics as a simple enough and complete enough equation to accurately describe CO_2_ and CH_4_ annual evolution. Moreover, the CO_2_ cycle was already fitted by a two-harmonic function and kernel calculations by Pérez et al. [50].

Fernández-Duque et al. [51] considered six kernel functions to fit both the trend and the annual evolution of CO_2_ and CH_4_. Although the Gaussian kernel provided low fits for the trend, agreement increased for the annual cycle. In this study, the relationship between observed and calculated values was satisfactory for both daily and annual cycles, as presented in Figure 6 and Figure 7 and in Table 3 and Table 4.

Pérez et al. [52] analysed the influence of four meteorological variables, boundary layer height, recirculation factor, wind direction, and wind speed, on the CO_2_ and CH_4_ annual cycle. Varied wind speed thresholds were used, and concentrations were analysed at night. Differences between average concentrations for observations above the highest threshold, 9 m·s^−1^, and all observations at night were around −1% for CO_2_ and CH_4_. However, when observations below the lowest threshold, 2 m·s^−1^, are selected, these differences are around 0.9 and 0.7%, for CO_2_ and CH_4_, respectively. The current analysis does not consider thresholds but rather wind speed intervals. The contrast between the average values calculated for the extreme intervals is around 2% for both gases.

## 5. Conclusions

The current study focuses on the relationship between CO_2_ and CH_4_ concentrations measured at a rural site and wind speed during nine years of observations.

Nine wind speed intervals were suggested, and the response of the mean CO_2_ concentrations revealed three groups: the first, below 4 m·s^−1^ with a slow decrease; the second, of wind speeds up to below 8 m·s^−1^, with a greater influence of wind speed; and the third, for wind speeds above 9 m·s^−1^, with concentrations that are only slightly greater than those of the interval 7–8 m·s^−1^, which could be attributed to background concentrations transported by wind. The concentration decrease with the increase in wind speed was more gradual for CH_4_ than for CO_2_.

Although concentration skewness was mainly positive due to outliers linked to stable stratification, close to zero or even negative skewness was observed in wind speed intervals above 6 m·s^−1^. These distributions may be only slightly affected by outliers owing to prevailing transport by wind, with the skewness contrast being noticeable for CH_4_.

Seven skewed distributions were used to fit the concentrations. Moreover, five efficiency criteria were considered. The most sensitive statistic was the modified Nash–Sutcliffe efficiency, with the correlation coefficient being the least sensitive. The Gumbel was the most suitable distribution function for CO_2_, followed by very similar fits for the gamma and beta distributions. The Weibull distribution presented the best fit for CH_4_. In contrast, the worst fit was for the exponential distribution.

Analysis of the daily cycle revealed the most frequent observations at midday for low wind speeds. Moreover, the daily cycle amplitude gradually decreased following the increase in wind speed, with the daily cycle being fairly slight for winds above 9 m·s^−1^.

Noticeable minima were observed in summer for both gases, with the drop in concentration being more marked for CO_2_. The contrast between the mean and median concentrations was noticeable for low wind speeds and could be attributed to the distribution shape and outlier influence. Although the mean concentration decreased with the increase in wind speed, the change in the annual cycle amplitude was less pronounced than that of the daily cycle. Moreover, CO_2_ amplitude was greater above 9 m·s^−1^ than below 2 m·s^−1^.

This research improves current knowledge of the relationship between wind speed and CO_2_ and CH_4_, although a more detailed analysis, such as the seasonal response of this relationship or directional studies, may be the subject of future research. In addition, combining observed and modelled measurements has proved successful and may be used when there is a sufficient number of observations, although its application with isolated measurements should be explored. Finally, these are promising research lines vis à vis obtaining better insights into the influence of meteorological variables on the concentrations of the two greenhouse gases studied.

## Figures and Tables

**Table 1:** Skewed distributions used in the current study.

Distribution	Probability Density Function	Parameter Calculation
Beta [11]	$f\left(x\right)=\left[\frac{\Gamma \left(\alpha +\beta \right)}{\Gamma \left(\alpha \right)\Gamma \left(\beta \right)}\right]x^{\alpha -1}{\left(1-x\right)}^{\beta -1};0\leq x\leq 1;\;\alpha ,\;\beta >0$	$\alpha =\frac{{\bar{x}}^{2}\left(1-\bar{x}\right)}{s^{2}}-\bar{x};\;\beta =\frac{\alpha \left(1-\bar{x}\right)}{\bar{x}};\;\bar{x}=\frac{\bar{y}-a}{b-a};$ $s_{x}^{2}=\frac{s_{y}^{2}}{{\left(b-a\right)}^{2}}$
Exponential [21]	f(x)=θ exp(−θ x);x>0; θ>0	$\theta =1/\bar{x}$
Frechet [22]	$f\left(x\right)=\frac{\lambda }{\sigma }{\left(\frac{\sigma }{x}\right)}^{\lambda +1}exp\left\{-{\left(\frac{\sigma }{x}\right)}^{\lambda }\right\};x\geq 0;\;\sigma ,\;\lambda >0$	−ln[−ln{F(x)}]=−λlnσ+λ ln(x)
Gamma [11]	$f\left(x\right)=\frac{{\left(x/\beta \right)}^{\alpha -1}exp\left(-x/\beta \right)}{\beta \;\Gamma \left(\alpha \right)};\;x,\alpha ,\;\beta >0$	$D=\ln\left(\bar{x}\right)-\frac{1}{n}\overset{n}{\underset{i=1}{\sum }}\ln\begin{array}{c} \left(x_{i}\right);\;\alpha =\frac{1+\sqrt{1+\frac{4D}{3}}}{4D};\;\\ \beta =\bar{x}/\alpha \end{array}$
Gumbel [11]	$f\left(x\right)=\frac{1}{\beta }exp\left\{-exp\left[-\frac{\left(x-\zeta \right)}{\beta }\right]-\frac{\left(x-\zeta \right)}{\beta }\right\}$	$\beta =\frac{x\sqrt{6}}{\pi };\;\zeta =\bar{x}-\gamma \beta ;\;\gamma =0.57721$
Lindley [21]	$f\left(x\right)=\frac{\theta^{2}}{\theta +1}\left(1+x\right)e^{-\theta x};x>0,\;\theta >0$	$\theta =\frac{-\left(\bar{x}-1\right)+\sqrt{{\left(\bar{x}-1\right)}^{2}+8\bar{x}}}{2\bar{x}}$
Weibull [23]	$f\left(x\right)=\left(\frac{\alpha }{\beta }\right){\left(\frac{x}{\beta }\right)}^{\alpha -1}exp\left[-{\left(\frac{x}{\beta }\right)}^{\alpha }\right];x,\;\alpha ,\;\beta >0$	ln(−ln(1−F(x)))=α ln(x)−α ln(β)

**Table 2:** Efficiency criteria.

Name	Equation
Pearson correlation coefficient	$r=\frac{\sum_{i=1}^{n}\left(O_{i}-\bar{O}\right)\left(C_{i}-\bar{C}\right)}{\sqrt{\sum_{i=1}^{n}{\left(O_{i}-\bar{O}\right)}^{2}}\sqrt{\sum_{i=1}^{n}{\left(C_{i}-\bar{C}\right)}^{2}}}$
Willmott index of agreement	$d=1-\frac{\sum_{i=1}^{n}{\left(O_{i}-C_{i}\right)}^{2}}{\sum_{i=1}^{N}{\left(\left|C_{i}-\bar{O}\right|+\left|O_{i}-\bar{O}\right|\right)}^{2}}$
Modified index of agreement	$d_{mod}=1-\frac{\sum_{i=1}^{n}\left|O_{i}-C_{i}\right|}{\sum_{i=1}^{N}\left(\left|C_{i}-\bar{O}\right|+\left|O_{i}-\bar{O}\right|\right)}$
Nash–Sutcliffe efficiency	$E=1-\frac{\sum_{i=1}^{n}{\left(O_{i}-C_{i}\right)}^{2}}{\sum_{i=1}^{n}{\left(O_{i}-\bar{O}\right)}^{2}}$
Modified Nash–Sutcliffe efficiency	$E_{mod}=1-\frac{\sum_{i=1}^{n}\left|O_{i}-C_{i}\right|}{\sum_{i=1}^{n}\left|O_{i}-\bar{O}\right|}$

**Table 3:** Statistics used to compare mean and modelled daily cycles.

	CO_2_		CH_4_	
	Cylindrical	Kernel	Cylindrical	Kernel
r	0.984	0.996	0.974	0.988
d	0.992	0.997	0.987	0.993
d_mod_	0.923	0.957	0.901	0.930
E	0.969	0.989	0.950	0.974
E_mod_	0.847	0.916	0.803	0.864

**Table 4:** Statistics used to compare mean and modelled annual cycles.

	CO_2_		CH_4_	
	Cylindrical	Kernel	Cylindrical	Kernel
r	0.923	0.990	0.890	0.984
d	0.944	0.982	0.919	0.980
d_mod_	0.814	0.915	0.802	0.919
E	0.828	0.940	0.774	0.937
E_mod_	0.646	0.840	0.630	0.848

## Data Availability

The CO_2_ and CH_4_ observations used in this study are available from the lead researcher of the project, M. Luisa Sánchez, upon request. METEX simulations were performed on http://db.cger.nies.go.jp/metex/trajectory.html (accessed on 28 July 2021).
